# An Improved Force Feedback Control Algorithm for Active Tendons

**DOI:** 10.3390/s120811360

**Published:** 2012-08-20

**Authors:** Tieneng Guo, Zhifeng Liu, Ligang Cai

**Affiliations:** School of Mechanical Engineering & Applied Electronics Technology, Beijing University of Technology, Beijing 100124, China; E-Mails: gtn@bjut.com (T.G.); cailigang1958@163.com (L.C.)

**Keywords:** vibration control, active tendon, differential force feedback

## Abstract

An active tendon, consisting of a displacement actuator and a co-located force sensor, has been adopted by many studies to suppress the vibration of large space flexible structures. The damping, provided by the force feedback control algorithm in these studies, is small and can increase, especially for tendons with low axial stiffness. This study introduces an improved force feedback algorithm, which is based on the idea of velocity feedback. The algorithm provides a large damping ratio for space flexible structures and does not require a structure model. The effectiveness of the algorithm is demonstrated on a structure similar to JPL-MPI. The results show that large damping can be achieved for the vibration control of large space structures.

## 1. Introduction

In general, large flexible space structures contain cable elements [1]. The cables are adopted to increase the stiffness of flexible structures, to maintain structure configuration, and to eliminate geometric uncertainty due to gaps. In recent years, a further step in exploiting the potential of cables has been proposed, whereby the cable acts as an active tendon to suppress structural vibrations. The idea was first presented for the vibration attenuation of bridges and buildings [2]. Researchers have introduced a lot of control algorithms for the active tendon. Leipholz and Abdel-Rohman [2] adopted a pole assignment method and optimal control method to attenuate the vibration of bridges and buildings. Betti and Panariello [3] adopted a linear quadratic regulator (LQR) to suppress the vibration of a building with a single degree of freedom (DOF). Chuang and Wu [4] extended the LQR to buildings with multi-DOFs to avoid structure destruction. Chang and Yu [5] proposed an optimal pole placement technique for the vibration control of a structure under a white noise ground excitation. Agrawal and Yang [6] investigated the performance of an optimal polynomial control method for the vibration suppression of a benchmark problem. Bani-Hani *et al.* [7] used a neural network control method for experimental vibration attenuation of a 1/4 scale model of a three-story steel frame. Kim and Yun [8] presented a sliding mode fuzzy control (SMFC) algorithm for vibration reduction of large structures. Lin *et al.* [9] adopted H_infinity_ optimality and H_2_ optimality to control the vibration of buildings with the soil-structure interaction (SSI) effect. Nudehi *et al.* [10] explored the use of end forces for vibration control in a cantilever beam with unilateral saturated nonlinear tendons. Then, Issa *et al.* [11] adopted this nonlinear tendon for vibration suppression in space structures.

**Figure 1.**
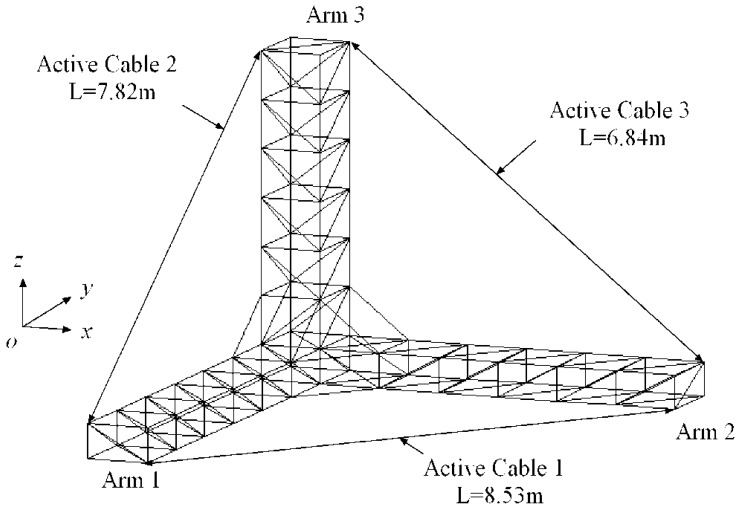
Guyed truss structure.

Most control schemes are based on an accurate model of the structure. However, when the model of the structure is not precisely defined or some active tendons fail, the performance of those control schemes deteriorates and can even cause instability of the control system. As a result, some studies have adopted decentralized schemes, which consist of independent controllers to activate specific tendons with only local feedback information. The decentralized schemes are useful for high dimensional large space structures, and can also be adapted to address the failure of some active tendons. Magana *et al.* [12] introduced a robust decentralized active control scheme based on the Lyapunov stability approach. Cao *et al.* [13] proposed a set of decentralized controllers that minimize the performance index of each subsystem to control the vibration of cable-stayed bridges subjected to vertical seismic excitation, Luo *et al.* [14] adopted a sliding mode decentralized controller to reduce structure vibrations by considering uncertainties in the parameters of stay cable geometry and unknown environmental excitation. Xu and Wu [15] proposed a decentralized non-parametric identification and control strategy with artificial neural networks for large-scale structures by using a neural network control scheme to reduce structure vibrations. Overall, these control schemes are complex and difficult to realize in practice. Preumont and colleagues [16–18] proposed an active tendon and integral force feedback algorithm to attenuate structure vibrations. The active tendon consists of a displacement actuator (piezoelectric actuator) and a co-located force sensor on the cable end. The preloaded active tendon is installed on the structure, as shown in Figure 1. By measuring the tension change *T* and controlling the active displacement, the vibration can be suppressed. The integral force feedback control algorithm is:

(1) $$u=-g\frac{T}{s}$$

where *g* is the integral feedback coefficient, *u* is the active displacement, and $\frac{1}{s}$ is the integral operator. Guo *et al.* [19] added a proportionality factor to the feedback law of Equation (1) and presented a proportional-integral (PI) algorithm to improve control effectiveness. The two control algorithms do not depend on the space structure model, and they are stable for the controlled structure. The control algorithm is simple, and the effectiveness of the algorithm is high. However, the achievable maximal damping ratio can be increased, especially for tendons with low axial stiffness.

This study presents a differential force feedback algorithm to suppress structure vibrations. The algorithm can introduce large active damping to structures. The kernel idea of the control algorithm is velocity feedback, so the stability of the control system can be guaranteed. The control algorithm is simple, and also, no structural model is required.

The paper is organized as follows: in Section 2, the governing equation of the structure is presented and the differential force feedback strategy is addressed. In Section 3, the stability and effectiveness of the control algorithm are discussed for when there are discrepancies between the actual axial stiffness of the tendon and the stiffness used for control law design. In Section 4, a simulation is demonstrated on a free-free structure similar to JPL-MPI [18], and the effectiveness of the control algorithm is investigated. Conclusions are drawn in Section 5.

## 2. Control Strategy

The mass of an active tendon in a large space structure is usually small compared with the mass of the truss structure. As a result, the dynamics of the active tendon can be restricted to the tension in the tendon in the vibration control and its interaction with the structure. A multi-tendon system is considered in the following deduction, where the number of tendons is *n*. The governing equation for the structure is:

(2) $$\text{M}s^{2}\text{x}+\text{Kx}=\sum_{i=1}^{n}{\text{b}}_{i}T_{i}$$

where ***M*** and ***K*** denote the structure mass and stiffness matrices without active tendons; *T_i_* is the tension on the *i*^th^ tendon, and ***b****_i_* is the influence matrix of the *i*^th^ tendon. The amount of damping on the large space structure is low, so it is neglected in the equation. Here, the mass of the tendon is small and neglected in the governing equation [16], therefore, the tendon can be considered as a spring. The tension in the *i*^th^ tendon is derived from the structure vibration and the tip displacement of the active tendon as follows:

(3) $$T_{i}=k_{ci}\left(u_{i}-{\text{b}}_{i}^{\text{T}}\text{x}\right),\;i=1,2,\cdots ,n$$

where *k_ci_* is the stiffness of the *i*^th^ active tendon, ***b****_i_*^T^***x*** is the relative displacement between the installation location of the *i*^th^ tendon on the space structure; and *u_i_* is the active displacement. Since the displacement actuator and the force sensor are co-located, the influence matrix ***b****_i_* is identical in Equations (2) and (3).

The control algorithm is the differential force feedback:

(4) $$u_{i}=k_{fi}\;\left(sT_{i}-k_{ci}su_{i}\right),\;i=1,2,\cdots ,n$$

where *k_fi_* is the feedback coefficient of the *i*^th^ active tendon. After eliminating the tension caused by the active displacement, the residual tension on the tendon is proportional to the vibration displacement. Therefore, the active displacement in Equation (4) is proportional to the velocity of the installation locations of the tendon, and the control algorithm increases the amount of damping to the structure. All controllable and observable states are asymptotically stable. From Equation (4), the active displacements of the *i*^th^ tendon can be expressed as follows:

(5) $$u_{i}=\frac{k_{fi}s}{k_{ci}k_{fi}s+1}T_{i},i=1,2,\cdots ,n$$

Eliminating *u_i_* between Equations (3) and (5), we obtain the tension on the tendon:

(6) $$T_{i}=-k_{fi}k_{ci}^{2}{\text{b}}_{i}^{\text{T}}s\text{x}-k_{ci}{\text{b}}_{i}^{\text{T}}\text{x},\;i=1,2,\cdots ,n$$

Substituting Equation (6) into governing Equation (2):

(7) $$\text{M}s^{2}\text{x}+\left(\sum_{i=1}^{n}k_{fi}k_{ci}^{2}{\text{b}}_{i}{\text{b}}_{i}^{\text{T}}\right)s\text{x}+\left(\text{K}+\sum_{i=1}^{n}k_{ci}{\text{b}}_{i}{\text{b}}_{i}^{\text{T}}\right)\text{x}=0$$

Obviously, if the feedback coefficient *k_fi_* > 0, then the tendon acts as the active damper. The amount of damping on the structure can be adjusted by modifying the feedback coefficient *k_fi_*. The stability of the control system is guaranteed.

## 3. Control Effectiveness

The differential force feedback algorithm (5) is simple and stable for controlled structures. It only depends on two coefficients. One is the feedback coefficient *k_fi_*, and the other is the tendon stiffness *k_ci_*, which can be measured. There is always some discrepancy between the actual tendon stiffness and the stiffness used for design of the control law. Therefore, it is necessary to analyze the stability and effectiveness of the control system when the error exists. Assuming that *k_ci_* + Δ*k_ci_* is used to design the control algorithm for the active tendon, *k_ci_* is the actual stiffness of the tendon, and Δ*k_ci_* is the error. The control law is described by:

(8) $$u_{i}=k_{fi}\;\left[sT_{i}-\left(k_{ci}+\Delta k_{ci}\right)su_{i}\right],i=1,2,\cdots ,n$$

Eliminating *u_i_* between Equations (3) and (8), we obtain the tension on the tendon:

(9) $$T_{i}=-k_{ci}{\text{b}}_{i}^{\text{T}}\text{x}-\frac{k_{ci}^{2}k_{fi}}{1+\Delta k_{ci}k_{fi}s}{\text{b}}_{i}^{\text{T}}s\text{x},\;i=1,2,\cdots ,n$$

Substituting Equation (9) into Equation (2), we can obtain the governing equation:

(10) $$\text{M}s^{2}\text{x}+\left(\text{K}+\sum_{i=1}^{n}k_{ci}{\text{b}}_{i}{\text{b}}_{i}^{\text{T}}\right)\text{x}+\sum_{i=1}^{n}\frac{k_{ci}^{2}k_{fi}}{1+\Delta k_{ci}k_{fi}s}{\text{b}}_{i}{\text{b}}_{i}^{\text{T}}s\text{x}=0$$

If Δ*k_ci_* > *0*, the control system is stable. The vibrations of the structure can be suppressed by the active tendon.

## 4. Numerical Results

**Figure 2.**
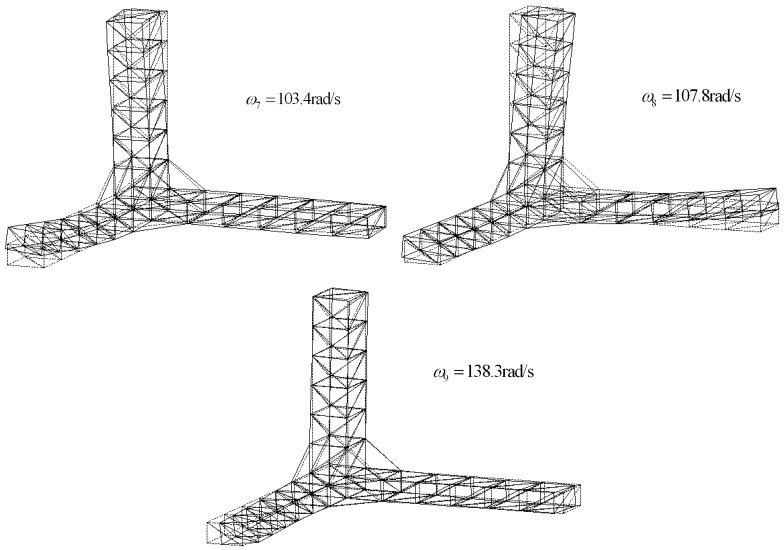
Mode shapes of the structure.

The free-free truss was adopted to assess the accuracy of the differential force feedback algorithm (Figure 1). The geometry is representative of the JPL-MPI [18]. The truss consists of aluminum pipes. The dimension of the structure and the parameters of the truss are shown in Table 1. The first three vibration modes without active tendons are displayed in Figure 2. In this study, two different types of tendon have been used: a soft tendon of 1 mm diameter made of polyethylene (*EA* = 4,000 *N*) and a stiffer one made of “Dynema” synthetic fiber (*EA* = 18,000 *N*) [18].

**Figure 3.**
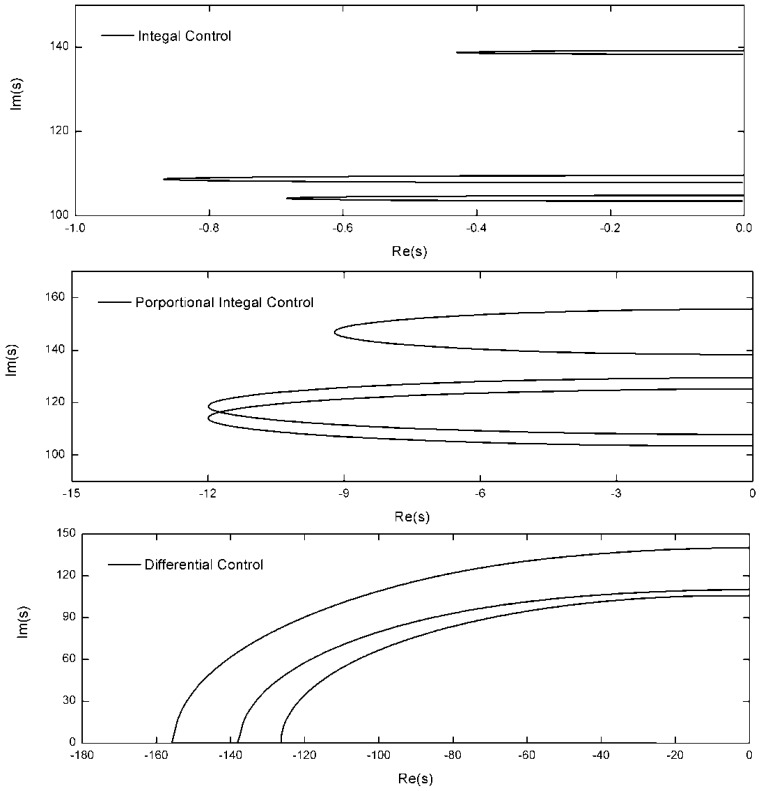
Root locus with three control strategies (*EA* = 4,000 *N*).

**Figure 4.**
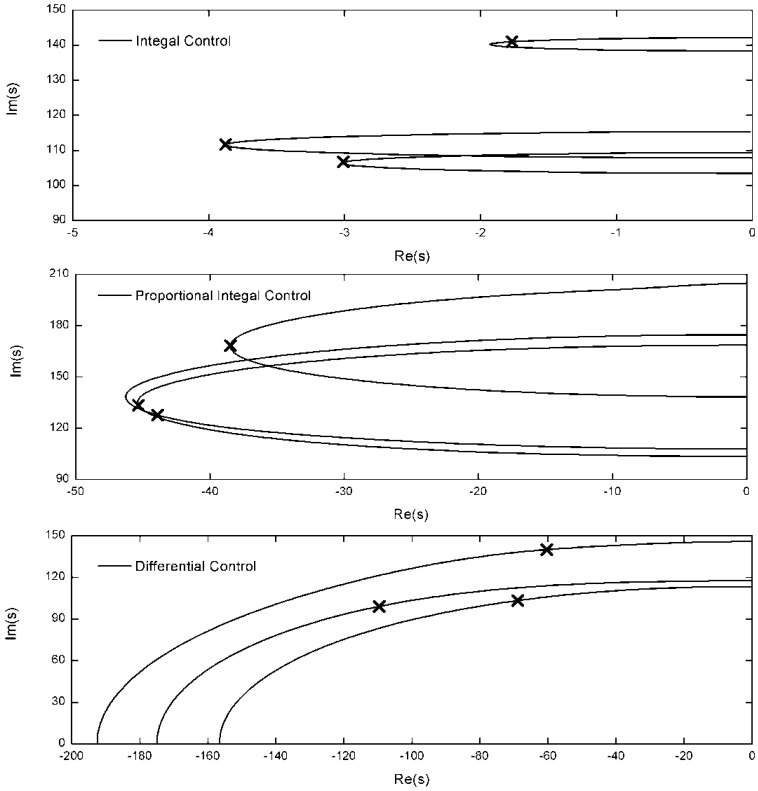
Root locus with three control strategies (*EA* = 18,000 *N*).

The preloaded tendons are installed on the tips of the structure. The natural frequencies of the three cables are greater than the control frequencies after adjustment of the preloaded tension. The integral force feedback, the PI control algorithm and the differential force feedback are adopted to control structure vibrations. The stiffness error of the active tendon is Δ*k_ci_* = 0.005*k_ci_*. For the JPL-MPI structure, the root-locus with respective to the feedback coefficients *g_i_* and *k_fi_* is shown in Figure 3 with the polyethylene tendon. According to the root-locus figure, the integral control can provide light damping for the structure. The PI control algorithm can provide more damping than the integral control. The damping obtained by the differential force feedback is greater than the other two control algorithm. The root-locus with the “Dynema” synthetic fiber is shown in Figure 4. Obviously, the damping obtained by three control algorithm is increased with the stiffer tendon. However, the damping obtained by the differential force feedback is also greater than the other two control algorithm. The differential force feedback in this study can provide a larger damping ratio for the structure for all soft tendon and stiff tendon cases.

**Figure 5.**
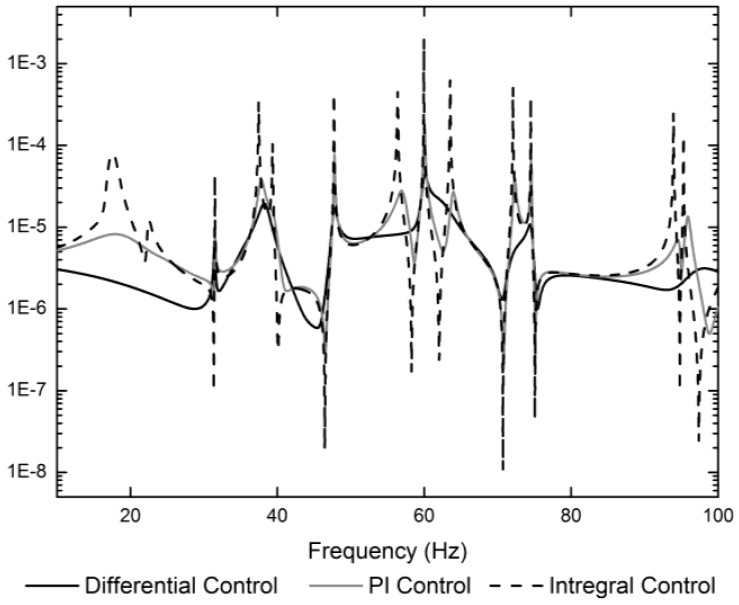
Frequency-response functions with three control strategies.

In order to compare the control effectiveness of the three control algorithm in the frequency region, a typical frequency-response function between the force applied in the middle of the truss and the displacement response on the top of arm 3 is shown in Figure 5 with three control algorithms. The active tendon is the synthetic fiber “Dynema” one. In fact, if the polyethylene tendon is adopted to attenuate the vibration, the comparative effectiveness of three control algorithm is more obvious. The integral feedback coefficient (*g_i_* = 0.044, *i* = 1, 2, 3), is selected to obtain the maximum damping ratio for the structure. The proportional integral feedback coefficient (the proportional coefficient *k_f_* = 1.1*k_c_*, the integral feedback coefficient *g_i_* = 0.0102, *i* = 1, 2, 3, are also selected to obtain the maximum damping ratio for the structure. The differential force feedback coefficients are set to *k_fi_* = 5.02 × 10^−5^ and the stiffness error is set to Δ*k_ci_* = 0.005*k_ci_, i* = 1, 2, 3. The × in Figure 4 marks the pole locations of the first three modes. The differential force feedback can provide a higher damping ratio than the integral force feedback and the PI control algorithm on both high order modes and low order modes.

**Figure 6.**
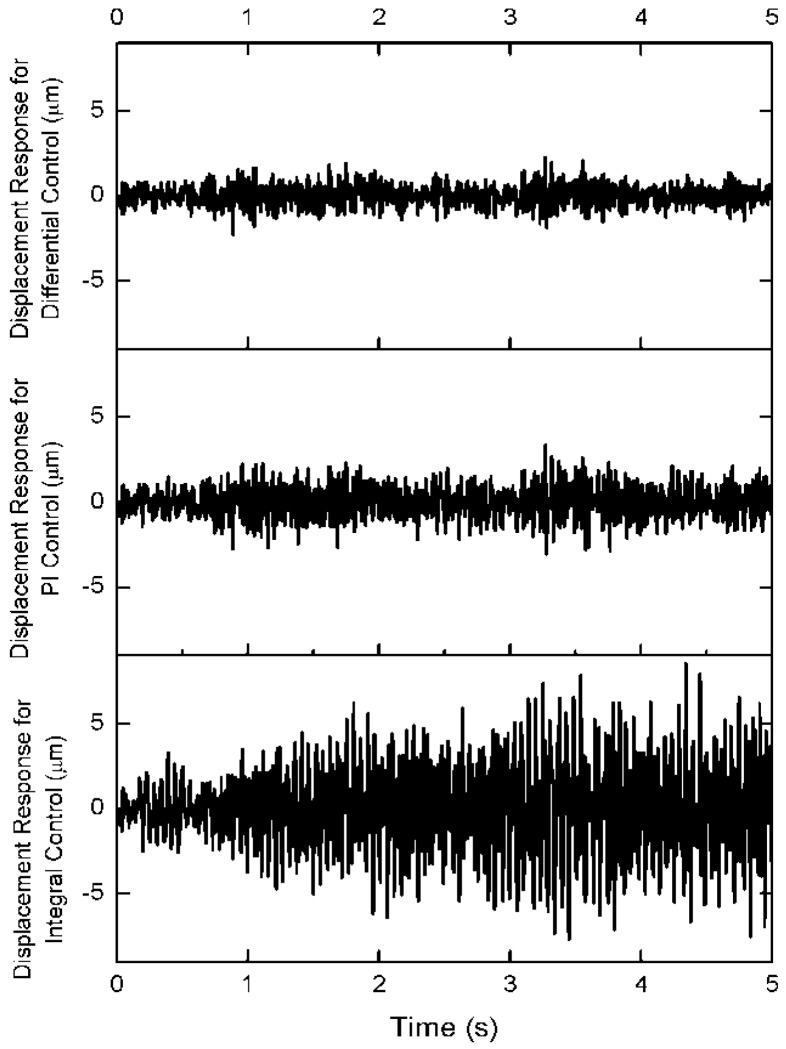
Displacement responses on the top of arm 3.

**Figure 7.**
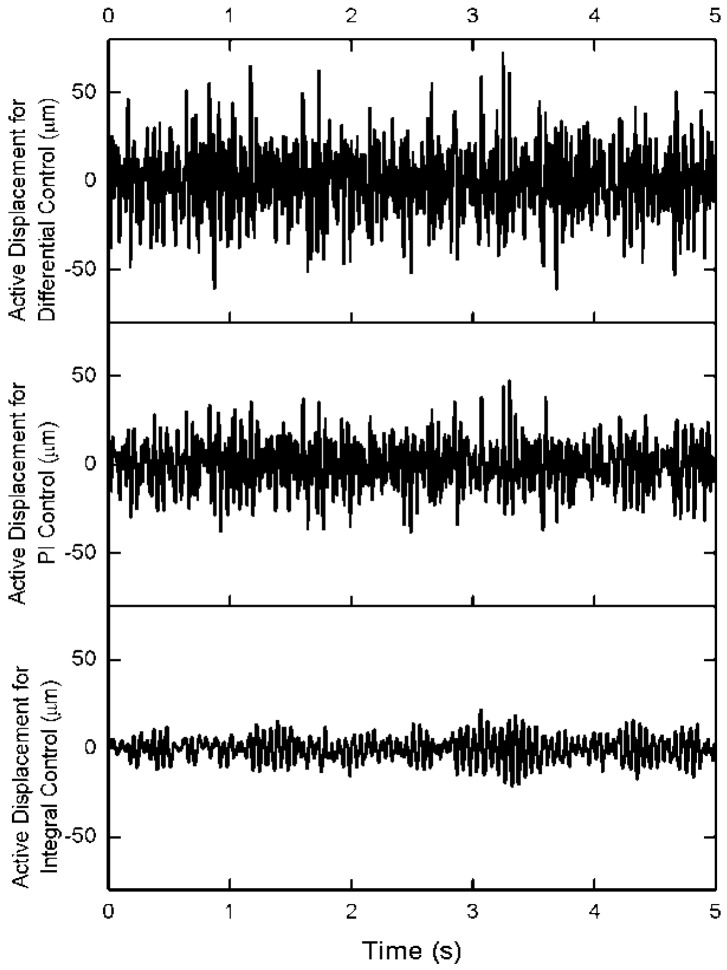
Active displacements on the cable 3.

In order to compare the control effectiveness in the time region, the three control algorithm is adopted to attenuate the truss vibration under the same while noise excitation in the middle of the truss. The coefficients of three control algorithm are selected as same as the coefficients on frequency-response curve. The displacement response on the top of structure is shown in Figure 6. The attenuation amplitude of structure vibration controlled by the differential force feedback scheme is lower than the PI control algorithm and the integral control algorithm. The active displacement is shown in Figure 7. The active displacement of the differential force feedback algorithm is larger slightly than the PI feedback algorithm.

The differential feedback can suppress effectively the structure vibrations for both lower order modes and high order modes. In order to obtain a high suppression rate, the large active displacement *u* is required. It is easy to comprehend this phenomenon. The lower attenuation amplitude of the controlled structure requires large control forces, especially for a tendon with low axial stiffness. Thus, it is convenient to set *k_fi_* to obtain a rational attenuation rate within the output range of actuators.

**Figure 8.**
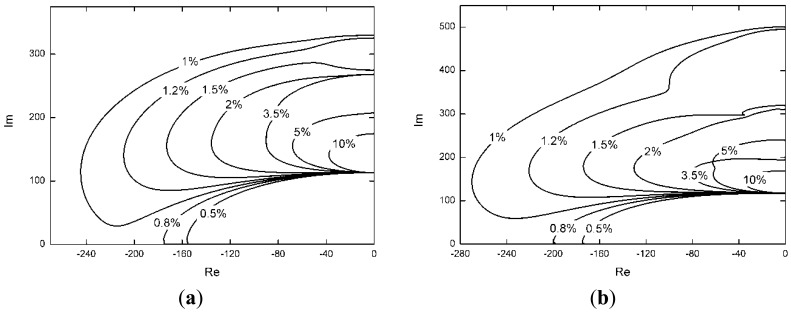

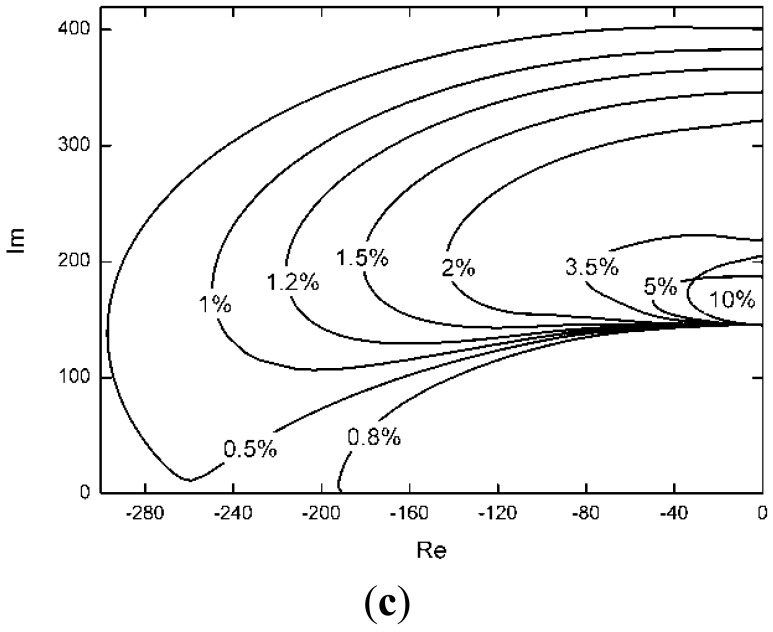
Root locus with different Δ*k_c_/k_c_*. (**a**) the first mode; (**b**) the second mode; (**c**) the third mode.

Because Δ*k_c_* is important in the control algorithm, the control effectiveness is investigated when there is a discrepancy between the actual stiffness of the tendon and the measured stiffness employed in the control law. If the stiffness of tendon changes, Δ*k_c_* will change, and the effectiveness of differential force feedback will also change. The root-locus of the control algorithm is shown under different Δ*k_c_/k_c_* in Figure 8. High damping can be obtained when Δ*k_c_/k_c_* is less than 2.5%.

**Figure 9.**
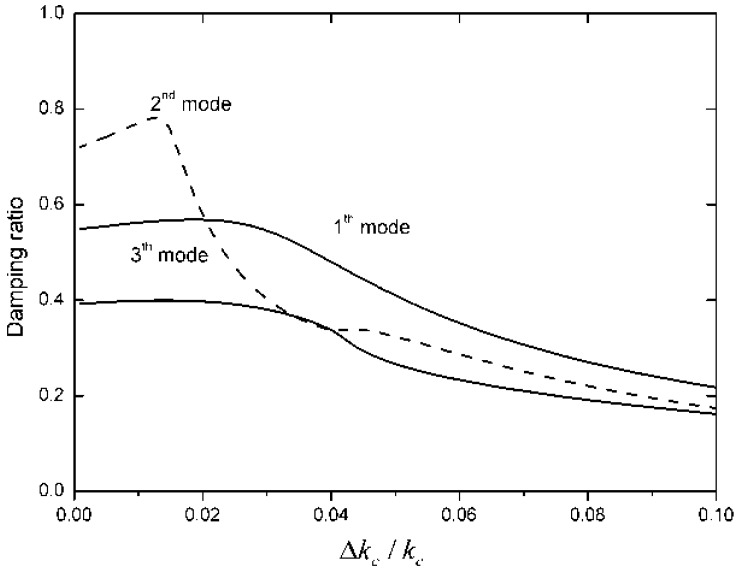
Relationship between damping ratio and error Δ*k_c_/k_c_*.

When the differential force feedback coefficients are set to *k_fi_* = 5.02 × 10^−5^, the curve of the achieved damping ratio and the resulting error is shown in Figure 9. Only first three order modes are determined. Although, the damping ratio of controlled structure varies as the error increases, high damping can be obtained when Δ*k_c_/k_c_* is less than 2.5%.

## 5. Conclusions

A differential force feedback algorithm to attenuate the vibration of large space structures was presented. The algorithm was based on the velocity feedback. The control algorithm did not require the structure model and the stability of the control system was guaranteed. The proposed control algorithm could achieve high damping ratios for structures. The effectiveness and stability of the differential force feedback algorithm was investigated accounting for discrepancies between the actual stiffness of the tendon and the stiffness used for the design of the control law. The simulation results for a free-free structure show that the proposed algorithm can provide more damping for the vibration suppression of a space structure.

## Figures and Tables

**Table 1.** Parameters of the space structure.

**Parameter**	**Value**
**Structure**	
Dimension of bays on arm 1	0.9 m × 1.1 m × 0.5 m
Dimension of bays on arm 2	0.9 m × 1.1 m × 0.5 m
Dimension of bays on arm 3	0.9 m × 1.1 m × 0.7 m
**Truss**	
Diameter	10 mm
Thickness	1 mm
Density	2,700 kg/m^3^
Young's Modulus	70 GPa
